# Supplementary material for: Androgen Receptor Gene CAG Repeat Length Varies and Affects Semen Quality in an Ethnic-Specific Fashion in Young Men from Russia
Source: Int J Mol Sci. 2022 Sep 13;23(18):10594. doi: 10.3390/ijms231810594 (PMC9505661; doi:10.3390/ijms231810594)
Supplement: Supplementary file 1 [file ijms-23-10594-s001.zip › ijms-1888605-supplementary.pdf]

Table S1. Frequency distribution of the CAG repeats in the entire Russian study population and subgroups with normal and impaired semen quality

No. of CAG repeats	Entire study population (n = 1313)		Normal semen quality (n = 739)		Impaired semen quality (n = 574)	
	No.	Frequency (%)	No.	Frequency (%)	No.	Frequency (%)
6	1	0.1	1	0.1		
7	1	0.1	1	0.1		
8	1	0.1			1	0.2
9						
10						
11	1	0.1	1	0.1		
12	1	0.1			1	0.2
13	1	0.1	1	0.1		
14	2	0.2	1	0.1	1	0.2
15	5	0.4	4	0.5	1	0.2
16	2	0.2			2	0.3
17	7	0.5	6	0.8	1	0.2
18	17	1.3	13	1.7	4	0.7
19	56	4.2	45	6.0	11	1.9
20	111	8.4	68	9.1	43	7.5
21	133	10.0	78	10.4	55	9.5
22	***207***	***15.6***	***115***	***15.4***	***92***	***15.9***
23	137	10.3	72	9.6	65	11.3
24	141	10.6	72	9.6	69	12.0
25	173	13.1	103	13.8	70	12.1
26	121	9.1	60	8.0	61	10.6
27	76	5.7	44	5.9	32	5.5
28	32	2.4	17	2.3	15	2.6
29	23	1.7	11	1.5	12	2.1
30	32	2.4	13	1.7	19	3.3

31	16	1.2	6	0.8	10	1.7
32	8	0.6	3	0.4	5	0.9
33	3	0.2	1	0.1	2	0.3
34	1	0.1			1	0.2
35						
36	3	0.2	3	0.4		
37						
38						
39	1	0.1			1	0.2

Note. No. - Number of each allele for a particular semen quality group; frequency - a percent of cases in relation to the respective number of participants within the group.

Table S2. Frequency distribution of the CAG repeats in the Slavic, Buryat and Yakut ethnic subgroups

No. of CAG repeats	Slavs (n = 697)		Buryats (n = 208)		Yakuts (n = 134)	
	n	Frequency (%)	n	Frequency (%)	n	Frequency (%)
6						
7	1	0.1				
8	1	0.1				
9						
10						
11			1	0.5		
12						
13						
14	1	0.1				
15	3	0.4				
16	1	0.1	1	0.5		
17	3	0.4	2	1.0		
18	11	1.6	2	1.0	1	0.7
19	38	5.4	12	5.7	1	0.7
20	77	10.9	9	4.3	4	3.0
21	86	12.2	21	10.0	4	3.0
**22**	***115***	***16.3***	***27***	***12.9***	16	11.9
23	75	10.7	22	10.5	13	9.7
24	75	10.7	24	11.5	13	9.7
**25**	80	11.4	23	11.0	***29***	***21.6***
26	51	7.2	22	10.5	23	17.2
27	29	4.1	17	8.1	12	9.0
28	15	2.1	7	3.3	4	3.0
29	7	1.0	6	2.9	6	4.5
30	19	2.7	5	2.4		
31	4	0.6	3	1.4	5	3.7

32	2	0.3	1	0.5	3	2.2
33	1	0.1				
34	1	0.1				
35						
36	1	0.1	2	1.0		
37						
38						
39			1	0.5		

Note. No. - Number of each allele for a particular semen quality group; frequency - a percent of cases in relation to the respective number of participants within the group.

Testing for AZF deletions of the Y-chromosome.

AZF deletions are direct causes of oligozoospermia or azoospermia. Identification of AZF deletions was performed using 13 STS markers and real-time multiplex PCR. Commercial kits “RealBest-Genetics AZF microdeletions” (“AO Vector-Best”, Novosibirsk) were used. The 13 STS markers were as follows: sY134, sY84, sY254, sY127, sY86, sY255, sY142, sY615, sY1191, sY1196, sY1206, sY1125, sY1296 (Figure).

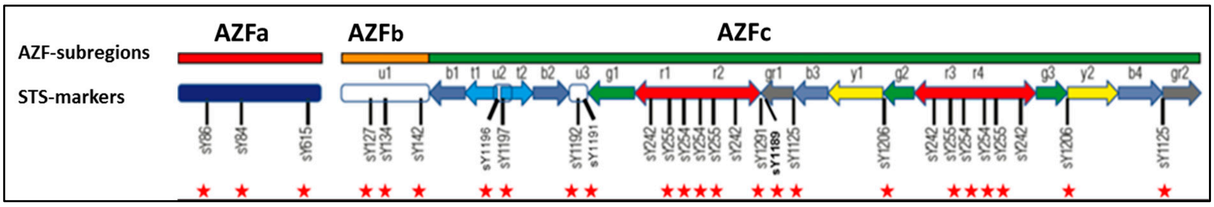

Figure S1. Schematic representation of the AZF locus and the main STS markers, an asterisk marks the STS markers used.
